# Supplementary material for: Relationship between Abnormal Placenta and Obstetric Outcomes: A Meta-Analysis
Source: Biomedicines. 2023 May 25;11(6):1522. doi: 10.3390/biomedicines11061522 (PMC10295197; doi:10.3390/biomedicines11061522)
Supplement: Supplementary file 1 [file biomedicines-11-01522-s001.zip › biomedicines-2300132-supplementary.pdf]

Supplemental File S1. The search strategy.

PubMed

#1 “abnormal placenta” [TIAB] OR “placenta abnormality” [TIAB] OR “placental variants” [TIAB]
#2 multilobed placenta [TIAB] OR bilobate placenta [TIAB] OR bilobed placenta [TIAB] OR placenta bilobate [TIAB] OR bipartite placenta [TIAB] OR placenta duplex [TIAB] OR succenturiate placenta [TIAB] OR accessory lobe [TIAB] OR succenturiate lobe [TIAB]OR circumvallate placenta [TIAB] OR circummarginate placenta [TIAB] OR fenestrated placenta [TIAB] OR ring-shaped placenta [TIAB] OR zonary placenta [TIAB] OR placenta membranacea
#3 #1 OR #2

Cochrane Library

#1 “abnormal placenta”: ab,ti,kw OR “placenta abnormality”: ab,ti,kw OR “placental variants”: ab,ti,kw
#2 multilobed placenta: ab,ti,kw OR placenta bilobate: ab,ti,kw OR bipartite placenta: ab,ti,kw OR placenta duplex: ab,ti,kw OR succenturiate lobe: ab,ti,kw OR fenestrated placenta: ab,ti,kw OR ring-shaped placenta: ab,ti,kw OR zonary placenta: ab,ti,kw OR placenta membranacea
#3 bilobate placenta: ab,ti,kw OR bilobed placenta: ab,ti,kw OR succenturiate placenta: ab,ti,kw OR accessory lobe: ab,ti,kw OR succenturiate lobe: ab,ti,kw OR circumvallate placenta: ab,ti,kw OR circummarginate placenta: ab,ti,kw OR fenestrated placenta: ab,ti,kw
#4 #1 OR #2 OR #3

Scopus

#1 TITLE-ABS-KEY (“abnormal placenta” OR “placenta abnormality” OR “placental variants”)
#2 TITLE-ABS-KEY (“multilobed placenta” OR “bilobate placenta” OR “bilobed placenta” OR “succenturiate placenta” OR “accessory lobe” OR “succenturiate lobe” OR “circumvallate placenta” OR “circummarginate placenta” OR fenestrated placenta)
#3 TITLE-ABS-KEY (“placenta bilobate” OR “bipartite placenta” OR “placenta duplex” OR “succenturiate lobe” OR “fenestrated placenta” OR “ring-shaped placenta” OR “zonary placenta” OR “placenta membranacea”)

(((TITLE-ABS-KEY ("abnormal placenta" OR "placenta abnormality" OR "placental variants")) OR (TITLE-ABS-KEY ("abnormal placenta" OR "placenta abnormality" OR "bilobate placenta" OR "bilobed placenta" OR "succenturiate placenta" OR "accessory lobe" OR "succenturiate lobe" OR "circumvallate placenta" OR "circummarginate placenta" OR fenestrated AND placenta)) OR (TITLE-ABS-KEY ("placenta bilobate" OR "bipartite placenta" OR "placenta duplex" OR "succenturiate lobe" OR "fenestrated placenta" OR "ring-shaped placenta" OR "zonary placenta" OR "placenta membranacea")))

Table S1. Metadata of eligible studies.

Author	Year	Location	Type	Placenta	Total	N	ART	CD	ID	PTB	PROM	HDP	FGR	IUFD	PA	PPH	PP	PAS	RPOC
Smith M [1]	2022	NZL	Case	Mul (Furcate)	--	1	--	--	--	--	--	--	○	--	--	--	--	--	--
Swanson K [2]	2021	USA	Origianl	Cir, Suc, Mul	311	11	--	--	--	--	--	--	--	--	--	--	--	--	--
Kutuk MS [3]	2021	TUR	Case	Mul	--	1	--	--	--	--	--	--	--	--	--	--	--	--	--
Celik OY [4]	2021	TUR	Original	Cir	154	77	--	○	--	○	--	○	--	○	○	--	--	--	--
Detweiler M [5]	2021	USA	Case	Suc	--	1	--	--	--	--	--	--	--	--	--	--	○	○	--
Volodarsky A [6]	2021	CAN	Original	Cir, Suc, Mul	677	25	○	--	--	--	--	--	--	--	--	--	--	--	--
Hasegawa J [7]	2021	JPN	Case	Cir	--	1	--	--	--	--	--	--	○	--	--	--	--	--	--
Sacha CR [8]	2020	USA	Original	Mul, Suc	1140	132	○	--	--	--	--	--	--	--	--	--	--	--	--
Volodarsky A [9]	2020	CAN	Original	Suc, Mul, Cir	1057	41	--	--	--	--	--	--	--	--	--	--	--	--	--
Abgral M [10]	2020	FRN	Case	Mul	--	2	--	--	--	--	--	--	--	--	--	--	--	--	--
Walter A [11]	2020	GER	Series	Mul	--	5	--	--	--	--	--	--	○	--	--	--	--	--	--
Tang L [12]	2019	CHN	Original	Mem	79862	3	--	--	--	--	○	--	○	○	--	--	○	--	--
Hamadeh S [13]	2018	KSA	Case	Suc	--	1	--	○	--	--	--	--	--	--	○	--	--	--	--
Sharma N [14]	2017	IND	Case	Cir	--	1	--	--	--	--	--	--	--	○	--	--	--	--	--
Stelzl PW [15]	2017	USA	Case	Suc	--	1	--	--	--	--	--	--	--	--	--	--	--	--	--
Ma JS [16]	2016	CHN	Original	Suc	28256	294	○	○	--	○	--	○	○	○	--	○	○	--	○
Biswas PR [17]	2016	BGD	Case	Mul	--	1	--	--	--	--	--	--	○	--	--	--	○	--	--
Yang PY [18]	2016	JPN	Case	Mul	--	1	--	--	--	--	--	--	--	--	--	--	--	--	--
Suzuki S [19]	2015	JPN	Original	Cir, Suc, Mul	16965	355	--	--	--	--	--	--	--	--	--	--	--	--	--
Patra S [20]	2015	IND	Case	Suc	--	1	--	--	--	--	--	--	--	--	--	○	○	--	--
Taniguchi H [21]	2014	JPN	Original	Cir	9149	92	○	○	○	○	○	--	--	--	○	--	--	--	--
Cavaliere AF [22]	2014	ITA	Case	Suc	--	1	--	○	--	--	--	--	--	--	--	--	--	--	--
Ravangard SF [23]	2013	USA	Case	Mem	--	1	--	--	--	○	--	--	--	--	○	--	--	--	--
Morales J [24]	2012	SPN	Case	Suc	--	1	--	--	--	--	--	--	--	--	--	--	○	--	--
Suzuki S [25]	2010	JPN	Original	Cir, Suc	11311	298	--	--	--	--	--	--	--	--	○	--	○	--	○
Suzuki S [26]	2008	JPN	Original	Cir	722	11	--	--	--	--	--	--	○	--	○	--	--	--	--
Suzuki S [27]	2008	JPN	Original	Cir	7930	139	○	○	○	○	--	○	○	○	○	○	--	--	--
Suzuki S [28]	2008	JPN	Original	Suc	7713	47	○	○	○	○	--	--	○	○	○	○	○	--	○
Seleye-Fubara D [29]	2005	NGA	Case	Suc	--	1	--	--	--	--	--	--	--	○	○	--	--	--	--
Ventolini G [30]	2004	USA	Original	Cir	88	4	--	--	--	--	--	--	--	--	--	--	--	--	--
Brucks UA [31]	2002	USA	Case	Mul	--	1	--	--	--	--	--	--	--	--	--	--	--	--	--
Shukunami K [32]	2001	JPN	Case	Suc	--	2	--	--	--	--	--	--	--	--	○	--	○	--	--
Walkup DWM [33]	2001	USA	Case	Mul	--	1	--	--	--	--		--	--	--	--	--	--	--	--
Chihara H [34]	2000	JPN	Case	Suc	--	1	--	--	--	--	--	--	--	--	--	--	--	--	--
Harris RD [35]	1997	USA	Original	Cir	--	13	--	--	--	○	--	○	○	--	○	--	--	--	--
Steemers NY [36]	1995	BEL	Case	Zonary	--	1	--	○	--	--	--	--	--	--	--	--	--	--	--
McCarthy J [37]	1995	USA	Original	CIr	--	12		○	--	--	--	--	--	--	○	○	--	--	--
Sistrom CL [38]	1993	USA	Original	Cir	1784	3	--	--	--	○	--	--	--	--	--	--	--	--	--
Dinh TV [39]	1992	USA	Case	Mem	--	1	--	--	--	--		--	--	--	--	--	--	○	--
Bey M [40]	1991	USA	Case	Cir	--	1	--	--	--	--	--	--	--	--	--	--	--	--	--
Greenberg JA [41]	1991	USA	Case	Mem	--	1	--	--	--	○	--	--	--	--	○	--	--	○	--

Wilkins BS [42]	1991	GER	Series	Mem	--	7	--	--	--	○	--	--	--	--	--	--	--	--	--
Hata K [43]	1988	JPN	Series	Suc	--	5	--	--	--	--	--	--	--	--	○	--	--	--	--
Jeanty P [44]	1983	BEL	Series	Suc	--	4	--	--	--	--	--	--	--	--	--	--	--	--	--
Rolschau J [45]	1978	DEN	Original	Cir	447	19	--	--	--	○	--	--	○	--	--	--	--	--	--
Nelson LH [46]	1977	USA	Case	Suc	--	1	--	--	--	○	--	--	--	--	--	--	--	--	--
Wladimiroff JW [47]	1976	NLD	Case	Mem	--	1	--	--	--	○	--	--	--	○	--	--	--	--	--
Mathews J [48]	1974	AUS	Case	Mem	--	1	--	--	--	○	--	--	--	--	--	--	--	--	--

Information regarding the outcome of interest was extracted and is summarized in Table. Abbreviations: ○, data is avaiable; N, number of women with circumvallate placenta; --, not applicable; NZL, New Zealand; USA, United States of America; TUR, Turkey; CAN, Canada; JPN, Japan; FRN, France; GER, Germany; CHN; China; KSA, Kingdom of Saudi Arabia; IND, India; BGD, Bangladesh; ITA, Italy; SPN, Spain; NGA, Nigeria; BEL, Belgium; DEN, Denmark; NLD, Netherland; AUS, Australia; ART, Assisted reproductive technology; CD, cesarean delivery; ID, instrumental delivery; PTB, preterm birth; PROM, preterm rupture of membrane; HDP, hypertensive disorder of pregnancy; FGR, fetal growth restriction; IUFD, intrauterine fetal death; PA, placenta abruption; PP, placenta previa; PPH, postpartum; PAS, placenta accreta spectrum and RPOC, retained products of conception.

Table S2. Risk bias assessment of comparator studies.

Authors	Confounding	Selection	Classification of intervention	Deviations from interventions	Missing data	Measurement of outcomes	Reported results	Overall bias
Celik OY [4]	● (serious)	● (serious)	● (serious)	● (no information)	● (no information)	● (moderate)	● (moderate)	● (serious)
Ma JS [16]	● (serious)	● (moderate)	● (serious)	● (no information)	● (no information)	● (moderate)	● (moderate)	● (serious)
Taniguchi H [21]	● (moderate)	● (moderate)	● (serious)	● (no information)	● (no information)	● (moderate)	● (moderate)	● (serious)
Suzuki S [25]	● (serious)	● (serious)	● (serious)	● (no information)	● (no information)	● (moderate)	● (moderate)	● (serious)
Suzuki S [26]	● (serious)	● (moderate)	● (serious)	● (no information)	● (no information)	● (moderate)	● (moderate)	● (serious)
Suzuki S [27]	● (serious)	● (moderate)	● (serious)	● (no information)	● (no information)	● (moderate)	● (moderate)	● (serious)
Rolschau J [45]	● (serious)	● (serious)	● (serious)	● (no information)	● (no information)	● (serious)	● (serious)	● (serious)

Risk of bias assessment was performed using the Risk Of Bias In Non-randomized Studies–of Interventions tool (ROBINS-I) [49-51].

● Low risk of bias (the study is comparable to a well-performed randomized trial with regard to this domain)

● Moderate risk of bias (the study is sound for a non-randomized study with regard to this domain but cannot be considered comparable to a well-performed randomized trial)

● Serious risk of bias (the study has some important problems in this domain)

● Critical risk of bias (the study is too problematic in this domain to provide any useful evidence on the effects of intervention.

● No information on how to base a judgment on the risk of bias for this domain.

Table S3. Study characteristics regarding the circumvallate placenta.

Author	Year	Location	Type	Comp	Placenta	Total	Control	N
Swanson K [2]	2021	USA	Original	--	Cir, Suc, Mul	311	--	3
Celik OY [4]	2021	TUR	Original	Yes	Cir	154	77	77
Volodarsky A [6]	2021	CAN	Original	No	Cir, Suc, Mul	677		8
Hasegawa J [7]	2021	JPN	Case	--	Cir	--	--	1
Volodarsky A [9]	2020	CAN	Original	--	Cir, Suc, Mul	1057	--	11
Sacha CR [8]	2020	USA	Original	--	Cir, Suc, Mul	1140	--	62
Sharma N [14]	2017	IND	Case	--	Cir	--	--	1
Suzuki S [19]	2015	JPN	Original	No	Cir, Suc, Mul	16965	16748	217
Taniguchi H [21]	2014	JPN	Original	Yes	Cir	9149	9057	92
Suzuki S [25]	2010	JPN	Original	Yes	Suc, Cir	11311	11096	215
Suzuki S [26]	2008	JPN	Original	Yes	Cir	722	711	11
Suzuki S [27]	2008	JPN	Original	Yes	Cir	7930	7666	139
Ventolini G [30]	2004	USA	Original	--	Cir	88	--	3
Harris RD [35]	1997	USA	Original	--	CIr	62	49	1*
McCarthy J [37]	1995	USA	Original	No	CIr	--	--	12
Sistrom CL [38]	1993	USA	Original	No	Cir	1784	1781	3
Bey M [40]	1991	USA	Case	--	Cir	--	--	1
Rolschau J [45]	1978	DEN	Original	Yes	Cir	447	426	19

*12 cases were partial circumvallate placenta. Abbreviations: type, type of study; comp, comparator study; Placenta, included type of abnormal placenta; --, not applicable; Mul, multilobed placenta, Suc, succenturiate placenta; Cir, circumvallate placenta; N, number of women with circumvallate placenta; Comp, comparator study; USA, United States of America; Tur, Turkey; CAN, Canada; JPN, Japan; DEN, Denmark

Table S4. Study characteristics regarding the succenturiate lobe placenta.

Author	Year	Location	Type	Placenta	Total	N
Swanson K [2]	2021	USA	Original	Suc, Mul, Cir	313	6
Detweiler M [5]	2021	USA	Case	Suc	--	1
Volodarsky A [6]	2021	CAN	Original	Suc, Mul, Cir	677	6
Sacha CR [8]	2020	USA	Original	Mul, Suc	1040	70
Volodarsky A [9]	2020	CAN	Original	Suc, Mul, Cir	1057	13
Hamadeh S [13]	2018	KSA	Case	Suc	--	1
Stelzl PW [15]	2017	USA	Case	Suc	--	1
Ma JS [16]	2016	CHN	Original	Suc	28256	294
Suzuki S [19]	2015	JPN	Original	Cir, Suc, Mul	16965	114
Patra S [20]	2015	IND	Case	Suc	--	1
Cavaliere AF [22]	2014	ITA	Case	Suc	--	1
Morales J [24]	2012	SPN	Case	Suc	--	1
Suzuki S [25]	2010	JPN	Original	Suc, Cir	11311	83
Suzuki S [28]	2008	JPN	Original	Suc	7703	47
Seleye-Fubara D [29]	2005	NGA	Case	Suc	--	1
Shukunami K [32]	2001	JPN	Case	Suc	--	2
Chihara H [34]	2000	JPN	Case	Suc	--	1
Hata K [43]	1988	JPN	Series	Suc	--	5
Jeanty P [44]	1983	BEL	Series	Suc	--	4
Nelson LH [46]	1977	USA	Case	Suc	--	1

Abbreviations: type, type of study; comp, complications; --, not applicable; Placenta, included type of abnormal placenta; Mul, multilobed placenta, Suc, succenturiate placenta; Cir, circumvallate placenta; N, number of women with succenturiate lobe placenta, USA, United States of America; CAN, Canada; KSA, Kingdom of Saudi Arabia; CHN, China; JPN, Japan; IND, India; ITA, Italy; NGA, Nigeria; BEL, Belgium.

Table S5. Study characteristics regarding the multilobed placenta.

Author	Year	Location	Type	Placenta	Total	N
Smith M [1]	2022	NZL	Case	Mul (Furcate)	--	1
Swanson K [2]	2021	USA	Original	Suc, Mul, Cir	311	2
Volodarsky A [6]	2021	CAN	Original	Suc, Mul, Cir	695	11
Volodarsky A [9]	2020	CAN	Original	Suc, Mul, Cir	1057	17
Abgral M [10]	2020	FRN	Case	Mul	--	2
Walter A [11]	2020	GER	Series	Mul	--	5*
Biswas PR [17]	2016	BGD	Case	Mul	--	1
Yang PY [18]	2016	JPN	Case	Mul	--	1
Suzuki S [19]	2015	JPN	Original	Cir, Suc, Mul	16965	24
Brucks UA [31]	2002	USA	Case	Mul	--	1
Walkup DWM [33]	2001	USA	Case	Mul	--	1

Abbreviations: type, type of study; comp, complications; Placenta, included type of abnormal placenta; Mul, multilobed placenta; Suc, succenturiate placenta; Cir, circumvallate placenta; N, number of women with multilobed placenta; --; not applicable; NZL, New Zealand; USA, United States of America; CAN, Canada; FRN, France; GER, Germany; BGD, Bangladesh; JPN, Japan. * All cases were twins.

Table S6. Previous studies regarding the placenta membranacea.

Author	Year	Location	Type	Total	N
Tang L [12]	2019	CHN	Original	79862	3
Ravangard SF [23]	2013	USA	Case	--	1
Dinh TV [39]	1992	USA	Case	--	1
Greenberg JA [41]	1991	USA	Case	--	1
Wilkins BS [42]	1991	GER	Series	--	7
Mathews J [48]	1974	AUS	Case	--	1
Wladimiroff JW [47]	1973	NLD	Case	--	1

Abbreviations: type, type of study; comp, complications; Placenta, included type of abnormal placenta; N, number of women with placenta membranacea; original, original articles, series, case series; case, case reports; --; not applicable; CHN, China; USA, United States of America; GER, Germany; AUS, Australia; NLD, Netherland.

References

1. Smith, M.; McCullum, B. Furcate umbilical cord insertion with a bilobed placenta identified on prenatal ultrasonography. *Australasian Journal of Ultrasound in Medicine* **2022**, *25*, 98-102, doi:https://doi.org/10.1002/ajum.12293.
2. Swanson, K.; Huang, D.; Kaing, A.; Blat, C.; Rosenstein, M.G.; Mok-Lin, E.; Gras, J.; Sperling, J.D. Is Preimplantation Genetic Testing Associated with Increased Risk of Abnormal Placentation After Frozen Embryo Transfer? *American journal of perinatology* **2021**, *38*, 105-110, doi:10.1055/s-0040-1714681.
3. Kutuk, M.S.; Takmaz, T.; Bayram, A.; Ozturk, S. Prenatal Sonographic Detection of Monochorionic Twins with Bipartite Placenta. *Fetal and pediatric pathology* **2021**, *40*, 685-690, doi:10.1080/15513815.2020.1725938.
4. Celik, O.Y.; Obut, M.; Keles, A.; Calık, M.G.; Dagdeviren, G.; Yucel, A.; Sahin, D. Outcomes of pregnancies diagnosed with circumvallate placenta, and use of uterine artery pulsatility index and maternal serum alpha-fetoprotein for prediction of adverse outcomes. *European journal of obstetrics, gynecology, and reproductive biology* **2021**, *262*, 57-61, doi:10.1016/j.ejogrb.2021.05.008.
5. Detweiler, M.; Downs, E. Sonographic Detection of Placenta Percreta With Associated Placenta Previa and Succenturiate Lobe. *Journal of Diagnostic Medical Sonography* **2021**, *37*, 194-199, doi:10.1177/8756479320978779.
6. Volodarsky-Perel, A.; Ton Nu, T.N.; Buckett, W.; Machado-Gedeon, A.; Cui, Y.; Shaul, J.; Dahan, M.H. Effect of embryo stage at transfer on placental histopathology features in singleton live births resulting from fresh embryo transfers. *Fertility and sterility* **2021**, *115*, 673-682, doi:10.1016/j.fertnstert.2020.06.017.
7. Hasegawa, J.; Furuya, N.; Doi, M.; Sasaki, T.; Takagi, M.; Suzuki, N. Sono-embryological assessments of a true knot that developed into a hypercoiled cord and circumvallate placenta. *The journal of maternal-fetal & neonatal medicine : the official journal of the European Association of Perinatal Medicine, the Federation of Asia and Oceania Perinatal Societies, the International Society of Perinatal Obstet* **2021**, *34*, 4162-4166, doi:10.1080/14767058.2019.1704247.
8. Sacha, C.R.; Harris, A.L.; James, K.; Basnet, K.; Freret, T.S.; Yeh, J.; Kaimal, A.; Souter, I.; Roberts, D.J. Placental pathology in live births conceived with in vitro fertilization after fresh and frozen embryo transfer. *American journal of obstetrics and gynecology* **2020**, *222*, 360.e361-360.e316, doi:10.1016/j.ajog.2019.09.047.
9. Volodarsky-Perel, A.; Nu, T.N.T.; Buckett, W.; Machado-Gedeon, A.; Cui, Y.; Shaul, J.; Dahan, M.H. Effect of newborn gender on placental histopathology and perinatal outcome in singleton live births following IVF. *Reproductive biomedicine online* **2020**, *41*, 907-916, doi:10.1016/j.rbmo.2020.08.002.
10. Abgral, M.; Martinovic, J.; Bonnin, A.; Houllier, M.; Senat, M.V.; Bouchghoul, H. Two separate placental masses on ultrasound do not always indicate a dichorionic pregnancy. *Journal of gynecology obstetrics and human reproduction* **2020**, *49*, 101694, doi:10.1016/j.jogoh.2020.101694.
11. Walter, A.; Strizek, B.; Berg, C.; Geipel, A.; Gembruch, U.; Engels, A.C. Outcome of monochorionic twins with prenatally diagnosed bipartite placenta. *Archives of gynecology and obstetrics* **2020**, *302*, 1549-1552, doi:10.1007/s00404-019-05364-w.
12. Tang, L.; Xu, L.; Hu, Y.; Zhang, L.; Wei, Q.; Wu, L. Placenta membranacea: an anormaly of the placenta: Three case reports. *Medicine* **2019**, *98*, e16166, doi:10.1097/md.0000000000016166.
13. Hamadeh, S.; Addas, B.; Hamadeh, N.; Rahman, J. Succenturiate placenta - abruption in a young primigravida: an unusual presentation. *CEOG* **2018**, *45*, 789-790, doi:10.12891/ceog4431.2018.
14. Sharma, N.; Das, R.; Salam, S.; Jethani, R.; Singh, A.S. Coexistent Circumvallate Placenta and Battledore Insertion of Umbilical Cord Resulting in Grave Obstetric Outcome: A Case Report. *Journal of reproduction & infertility* **2017**, *18*, 390-392.
15. Stelzl, P.W.; Yadav, G.; Perley, L.; Silasi, M. Early sonographic detection of a succenturiate placenta after IVF in a 42-year-old woman with multiple comorbidities. *BMJ case reports* **2017**, *2017*, doi:10.1136/bcr-2017-222189.
16. Ma, J.S.; Mei, X.; Niu, Y.X.; Li, Q.G.; Jiang, X.F. Risk Factors and Adverse Pregnancy Outcomes of Succenturiate Placenta: A Case-Control Study. *The Journal of reproductive medicine* **2016**, *61*, 139-144.
17. Biswas, P.R.; Paul, G.K.; Chowdhury, M.; Selim, M. A Rare Shape of Placenta; Placenta Bipartite, Bilobata, Dimidiate, Duplex: A Rare Case Report. *Bangladesh Journal of Obstetrics & Gynaecology* **2017**, *31*, 101-103, doi:10.3329/bjog.v31i2.34220.
18. Yang P-Y, K.K., Ishibashi M, Mori N, Hata T. . HDliveFlow with HDlive Silhouette Mode in Antenatal Diagnosis of Bilobed Placenta. *Donald School J Ultrasound Obstet Gynecol* **2016**, *10*, 415-417.
19. Suzuki, S.; Kato, M. Clinical Significance of Pregnancies Complicated by Velamentous Umbilical Cord Insertion Associated With Other Umbilical Cord/Placental Abnormalities. *Journal of clinical medicine research* **2015**, *7*, 853-856, doi:10.14740/jocmr2310w.
20. Patra, S.; Mistry, P.K.; Dutta, M.; Das, I. Placenta Previa with Succenturiate Placenta◎Delivered with a Living Healthy Baby. *Bangladesh Journal of Obstetrics & Gynaecology* **2016**, *30*, 116-118, doi:10.3329/bjog.v30i2.30908.
21. Taniguchi, H.; Aoki, S.; Sakamaki, K.; Kurasawa, K.; Okuda, M.; Takahashi, T.; Hirahara, F. Circumvallate placenta: associated clinical manifestations and complications-a retrospective study. *Obstetrics and gynecology international* **2014**, *2014*, 986230, doi:10.1155/2014/986230.
22. Cavaliere, A.F.; Rosati, P.; Ciliberti, P.; Buongiorno, S.; Guariglia, L.; Scambia, G.; Tintoni, M. Succenturiate lobe of placenta with vessel anomaly: a case report of prenatal diagnosis and literature review. *Clinical imaging* **2014**, *38*, 747-750, doi:10.1016/j.clinimag.2014.01.018.
23. Ravangard, S.F.; Henderson, K.; Fuller, K. Placenta membranacea. *Archives of gynecology and obstetrics* **2013**, *288*, 709-712, doi:10.1007/s00404-013-2778-z.
24. Morales-Roselló, J.; Peralta Llorens, N. Doppler impedance changes at the fetal brain vessels in a pregnancy affected with a multiple combination of uteroplacental anomalies. *Case reports in medicine* **2012**, *2012*, 293156, doi:10.1155/2012/293156.
25. Suzuki, S.; Igarashi, M.; Inde, Y.; Miyake, H. Abnormally shaped placentae in twin pregnancy. *Archives of gynecology and obstetrics* **2010**, *281*, 65-69, doi:10.1007/s00404-009-1064-6.
26. Suzuki, S. Antenatal screening for circumvallate placenta. *Journal of medical ultrasonics (2001)* **2008**, *35*, 71-73, doi:10.1007/s10396-007-0168-5.
27. Suzuki, S. Clinical significance of pregnancies with circumvallate placenta. *The journal of obstetrics and gynaecology research* **2008**,

34, 51-54, doi:10.1111/j.1447-0756.2007.00682.x.

28. Suzuki, S.; Igarashi, M. Clinical significance of pregnancies with succenturiate lobes of placenta. *Archives of gynecology and obstetrics* **2008**, *277*, 299-301, doi:10.1007/s00404-007-0482-6.
29. Seleye-Fubara, D.; Akani, C.I. Succenturiate placenta: a rare variant in Rivers State Nigeria (a report of one case). *Nigerian journal of medicine : journal of the National Association of Resident Doctors of Nigeria* **2005**, *14*, 325-326.
30. Ventolini, G.; Samlowski, R.; Hood, D.L. Placental findings in low-risk, singleton, term pregnancies after uncomplicated deliveries. *American journal of perinatology* **2004**, *21*, 325-328, doi:10.1055/s-2004-831882.
31. Brucks, U.A.; Duval, J.R. Perinatal Evaluation of the Bilobed or Bipartite Placenta. *Journal of Diagnostic Medical Sonography* **2002**, *18*, 161-166, doi:10.1177/87579302018003009.
32. Shukunami, K.; Tsunezawa, W.; Hosokawa, K.; Tajima, K.; Kotsuji, F. Placenta previa of a succenturiate lobe: a report of two cases. *European journal of obstetrics, gynecology, and reproductive biology* **2001**, *99*, 276-277, doi:10.1016/s0301-2115(01)00385-2.
33. Walkup, D.W.M. A Rare Case of Duplicated Placenta and Bifurcated Umbilical Cord in a Singleton Pregnancy. *Journal of Diagnostic Medical Sonography* **2001**, *17*, 280-285, doi:10.1177/875647930101700507.
34. Chihara, H.; Otsubo, Y.; Ohta, Y.; Araki, T. Prenatal diagnosis of succenturiate lobe by ultrasonography and color Doppler imaging. *Archives of gynecology and obstetrics* **2000**, *263*, 137-138, doi:10.1007/s004040050013.
35. Harris, R.D.; Wells, W.A.; Black, W.C.; Chertoff, J.D.; Poplack, S.P.; Sargent, S.K.; Crow, H.C. Accuracy of prenatal sonography for detecting circumvallate placenta. *AJR. American journal of roentgenology* **1997**, *168*, 1603-1608, doi:10.2214/ajr.168.6.9168736.
36. Steemers, N.Y.; De Rop, C.; Van Assche, A. Zonary placenta. *International journal of gynaecology and obstetrics: the official organ of the International Federation of Gynaecology and Obstetrics* **1995**, *51*, 251-253, doi:10.1016/0020-7292(95)80020-4.
37. McCarthy, J.; Thurmond, A.S.; Jones, M.K.; Sistrom, C.; Scanlan, R.M.; Jacobson, S.L.; Lowensohn, R. Circumvallate placenta: sonographic diagnosis. *Journal of ultrasound in medicine : official journal of the American Institute of Ultrasound in Medicine* **1995**, *14*, 21-26, doi:10.7863/jum.1995.14.1.21.
38. Sistrom, C.L.; Ferguson, J.E. Abnormal membranes in obstetrical ultrasound: incidence and significance of amniotic sheets and circumvallate placenta. *Ultrasound in obstetrics & gynecology : the official journal of the International Society of Ultrasound in Obstetrics and Gynecology* **1993**, *3*, 249-255, doi:10.1046/j.1469-0705.1993.03040249.x.
39. Dinh, T.V.; Bedi, D.G.; Salinas, J. Placenta membranacea, previa and accreta. A case report. *The Journal of reproductive medicine* **1992**, *37*, 97-99.
40. Bey, M.; Dott, A.; Miller, J.M., Jr. The sonographic diagnosis of circumvallate placenta. *Obstetrics and gynecology* **1991**, *78*, 515-517.
41. Greenberg, J.A.; Sorem, K.A.; Shifren, J.L.; Riley, L.E. Placenta membranacea with placenta increta: a case report and literature review. *Obstetrics and gynecology* **1991**, *78*, 512-514.
42. Wilkins, B.S.; Batcup, G.; Vinall, P.S. Partial placenta membranacea. *British journal of obstetrics and gynaecology* **1991**, *98*, 675-679, doi:10.1111/j.1471-0528.1991.tb13454.x.
43. Hata, K.; Hata, T.; Aoki, S.; Takamori, H.; Takamiya, O.; Kitao, M. Succenturiate placenta diagnosed by ultrasound. *Gynecologic and obstetric investigation* **1988**, *25*, 273-276, doi:10.1159/000293798.
44. Jeanty, P.; Kirkpatrick, C.; Verhoogen, C.; Struyven, J. The Succenturiate Placenta. *Journal of Ultrasound in Medicine* **1983**, *2*, 9-12, doi:https://doi.org/10.7863/jum.1983.2.1.9.
45. Rolschau, J. CIRCUMVALLATE PLACENTA AND INTRAUTERINE GROWTH RETARDATION. *Acta obstetricia et gynecologica Scandinavica* **1978**, *57*, 11-14, doi:10.1111/aogs.1978.57.s72.11.
46. Nelson, L.H.; Fishburne, J.I.; Stearns, B.R. Ultrasonographic description of succenturiate placenta. *Obstetrics and gynecology* **1977**, *49*, 79-80.
47. Wladimiroff, J.W.; Wallenburg, H.C.; vanden Putten, P.; Drogendijk, A.C. Ultrasonic diagnosis of placenta membranacea. *Archiv fur Gynakologie* **1976**, *221*, 167-174, doi:10.1007/bf00667146.
48. Mathews, J. Placenta Membranacea. *Australian and New Zealand Journal of Obstetrics and Gynaecology* **1974**, *14*, 45-47, doi:https://doi.org/10.1111/j.1479-828X.1974.tb00823.x.
49. Sterne, J.A.; Hernan, M.A.; Reeves, B.C.; Savovic, J.; Berkman, N.D.; Viswanathan, M.; Henry, D.; Altman, D.G.; Ansari, M.T.; Boutron, I.; et al. ROBINS-I: a tool for assessing risk of bias in non-randomised studies of interventions. *BMJ* **2016**, *355*, i4919, doi:10.1136/bmj.i4919.
50. Danna, S.M.; Graham, E.; Burns, R.J.; Deschenes, S.S.; Schmitz, N. Association between Depressive Symptoms and Cognitive Function in Persons with Diabetes Mellitus: A Systematic Review. *PLoS One* **2016**, *11*, e0160809, doi:10.1371/journal.pone.0160809.
51. ROBINS-I detailed guidance (2016). https://www.riskofbias.info/welcome/home/current-version-of-robins-i/robins-i-detailed-guidance-2016. (accessed 02/20/2023.
